# Empowerment as the key to health-enhancing physical activity in patients with rheumatoid arthritis and low physical function—a cross-sectional study

**DOI:** 10.1007/s10067-025-07696-2

**Published:** 2025-11-03

**Authors:** M. L. E. Andersson, K. Wibring, S. Bergman, A. Bremander

**Affiliations:** 1 Department of Clinical Sciences, Section of Rheumatology, Faculty of Medicine, Lund University, Lund, Sweden; 2 Spenshult Research and Development Center, Halmstad, Sweden; 3 Department of Environmental and Biosciences School of Business, Innovation and Sustainability, Halmstad University, Halmstad, Sweden; 4 Department of Rheumatology, Capio Movement, Halmstad, Sweden; 5 Primary Health Care, School of Public Health and Community Medicine, Institute of Medicine, Sahlgrenska Academy, University of Gothenburg, Gothenburg, Sweden; 6 Department of Regional Health Research, University of Southern Denmark, Odense, Denmark; 7 Danish Hospital for Rheumatic Diseases, University Hospital of Southern Denmark, Sønderborg, Denmark

**Keywords:** Empowerment, Physical activity, Physical function, Rheumatoid arthritis

## Abstract

**Objective:**

To study factors associated with health-enhancing physical activity in people with rheumatoid arthritis (RA), stratified by physical function.

**Method:**

In 2017, a survey was sent to 1543 patients with RA in the BARFOT (Better Anti-Rheumatic Pharmacotherapy) cohort, and 69% of patients responded. The survey included questions on physical function, pain, fatigue, self-reported disease activity, physical activity level, health-related quality of life, empowerment, comorbidities, and antirheumatic treatment. The patients were stratified based on physical function according to the Health Assessment Questionnaire (median value as cut off) into groups with worse vs. better physical function and further dichotomized to whether or not they met the World Health Organisation recommended level of health-enhancing physical activity (≥ 150 min/week). The Mann–Whitney *U* test or chi-squared test was used to analyse group differences. A logistic regression model adjusted for age and sex was used to study factors associated with health-enhancing physical activity.

**Results:**

In total, 1047 patients had available data on physical function, mean age 67 years (SD 13), 72% women. Younger age, non-obesity, less pain, less fatigue, and lower disease activity were associated with health-enhancing physical activity, irrespective of physical function level. In the group with worse physical function, better health-related quality of life and empowerment were also associated with health-enhancing physical activity.

**Conclusion:**

The results indicate a complex relationship between various factors that can affect the level of physical activity in individuals with RA. Strengthening empowerment could be a key when supporting improvement in physical activity in patients with RA with impaired physical function.

**Table:** 

Key Points
• *The importance of empowerment in managing rheumatoid arthritis*.
• *The role of physical activity in improving physical function*.
• *Strategies for integrating physical activity into daily life for those with rheumatoid arthritis*.

## Introduction

Rheumatoid arthritis (RA) is a chronic inflammatory joint disease that impacts everyday life for the patients [1]. Limiting factors in everyday life can include pain, fatigue, stiffness, and reduced joint mobility [1]. Patients with RA have impaired physical function and worse health-related quality of life, compared to the general population [2]. It is well known that physical activity positively affects health in general and recommendations for health-enhancing physical activity (HEPA) in patients with RA are similar to those for the general population. The facilitation of healthy levels of physical activity should be incorporated into standard care throughout the disease course for patients with RA, considering individual limitations [3, 4]. 

Studies on patients with RA have shown that physical activity positively affects both systemic manifestations of RA, such as increased cardiovascular risk [5], and various disease-related symptoms, such as swollen and tender joints [6]. Improvements in physical function, reduced fatigue, and reduced risk of arthritis-related joint damage have also been reported in patients engaging in physical activity [6–9]. Despite all the potential positive effects, people with RA are less physically active than the general population [10].

The overall relationship between physical function and physical activity is well known. However, it is essential to study factors associated with physical activity in patients with varying physical challenges to support the delivery of person-centred care. Diversified clinical support might be required for the range of patients with physical challenges to improve or maintain their physical activity level. Hence, the aim was to study factors associated with fulfilling HEPA in people with RA, stratified by physical functioning.

## Method

### Design

**Fig. 1:**
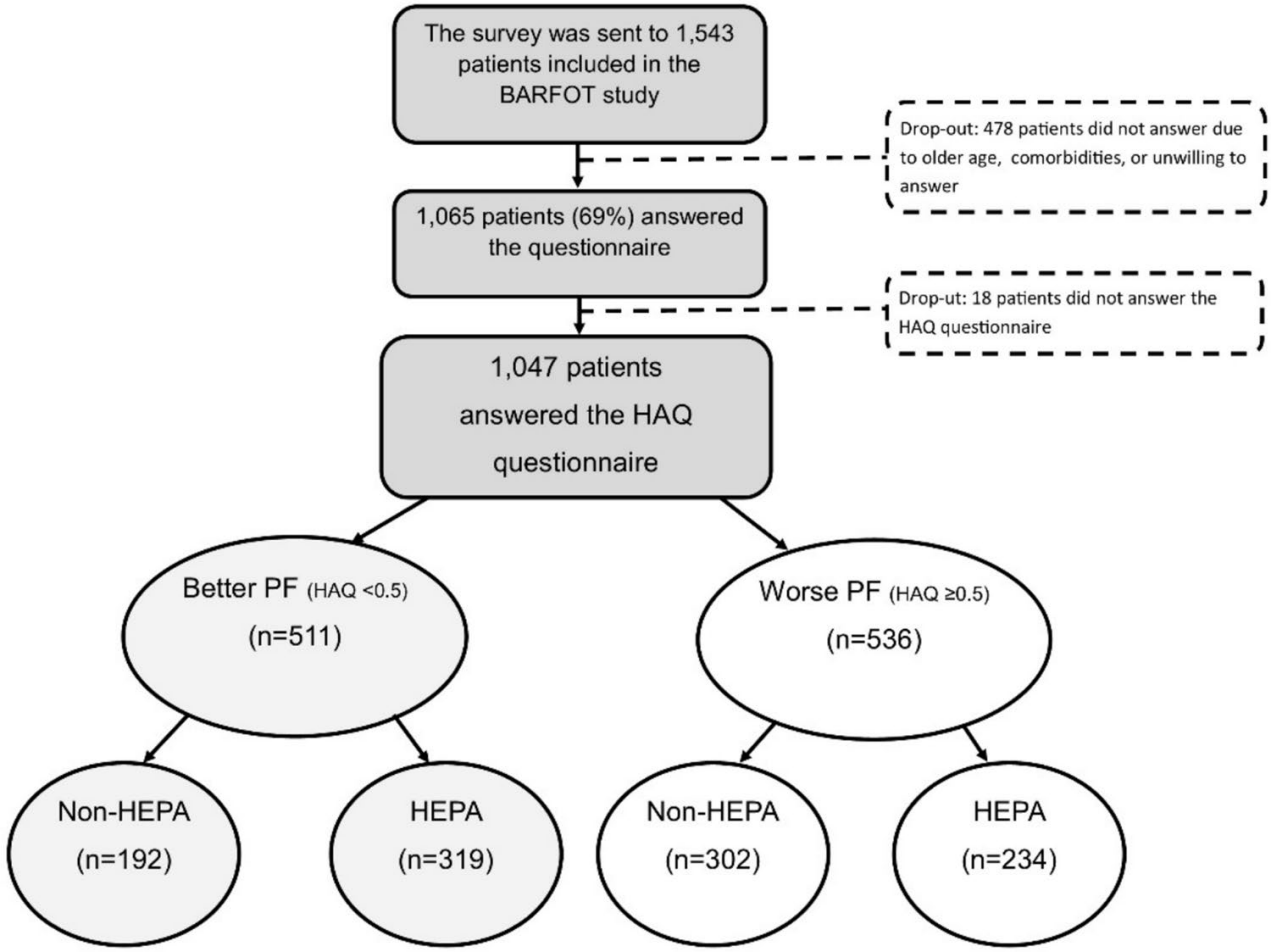
Flowchart of the study showing the dichotomised groups by health assessment questionnaire (HAQ), better physical function (PF), worse PF, and those who fulfilled the recommended level of health-enhancing physical activity (HEPA) or not (Non-HEPA) in the separate groups

This cross-sectional study included patients from the Better Anti-Rheumatic Pharmacotherapy (BARFOT) study. BARFOT is a longitudinal, multicentre, observational study of patients with early RA. The inclusion started in 1992 and was completed in 2006 [11]. Patients were consecutively included after being diagnosed with RA, according to the American College of Rheumatology 1987 criteria [12], with a disease duration of ≤ 12 months at inclusion. In 2017, a postal survey was sent to 1543 eligible patients, of which 1065 (69%) patients responded. All patients who, in the 2017 survey, had answered the Health Assessment Questionnaire (HAQ)—which measures physical function—were included in this study (Fig. 1).

### The survey

The survey included valid and commonly used questionnaires: the HAQ (0–3, best to worst) [13], EuroQol 5 dimensions with three response options (EQ5D-3L; 0–1, worst to best) [14], and the Swedish Rheumatic Disease Empowerment scale (SWE-RES-23; 1–5, worst to best) [15]. The SWE-RES-23 questionnaire includes 23 items, in five subscales. Subscale 1 concerns goal achievement and overcoming barriers to goal achievement; subscale 2—self-knowledge, subscale 3—managing stress, subscale 4—assessing dissatisfaction and readiness to change, and subscale 5—support for caring. Physical activity was assessed by questions concerning frequency and duration, which were reported as minutes/week spent on moderate and vigorous physical activity. The questions adhered to Swedish population studies on lifestyle habits (only available in Swedish, https://www.socialstyrelsen.se/globalassets/sharepointdokument/dokument-webb/nationella-riktlinjer/levnadsvanor-fragor-om-levnadsvanor.pdf). Physical activity was dichotomised to whether or not they fulfilled HEPA (being physically active ≥ 150 min per week on a moderate level or ≥ 75 min per week on a vigorous level) [3].

The survey also included questions about pain, fatigue, patient global assessment, assessed by a numeric rating scale (NRS, 0–10, best to worst), the number of swollen and tender joints according to 28-joint disease activity score (DAS28) [16], and comorbidities. Responses on the presence of comorbidities were yes/no and included cardiovascular disease, pulmonary disease, diabetes type 1, diabetes type 2, fibromyalgia, Mb. Crohn, psoriasis, and depression. The survey also included self-reported medical treatment, smoking habits, height, weight, and waist circumference. The medical treatments were grouped into conventional disease-modifying antirheumatic drugs (cDMARD), biological DMARD (bDMARD), and corticosteroids (CS). Smoking was dichotomised as never or ever smokers. Body mass index (BMI) was calculated in four groups: underweight BMI ≤ 18.4 kg/m^2^, normal weight BMI 18.5–24.99 kg/m^2^, overweight BMI 25–29.9 kg/m^2^, and obese BMI ≥ 30 kg/m^2^.

### Statistics

The patients were stratified into two groups by the median value of HAQ, where HAQ < 0.5 represents better physical function, and HAQ ≥ 0.5 represents worse physical function. In each group, patients who reported HEPA were compared to those who did not. The chi-squared test was used for proportions and the Mann–Whitney *U* test for continuous variables to test differences between groups due to skewness in the distribution of data. Bivariate logistic regression was used to analyse associations with HEPA in the two groups stratified by PF. The multivariate model was adjusted for age and sex. Variables with a *P*-value of ≤ 0.25 in the univariate analysis were included in a multivariate model [17]. All significance testing was two-tailed and conducted at the 0.05 significance level. Statistical analyses were performed using SPSS Statistics version 29 software (IBM, New York, US).

### Ethical statement

The postal survey received ethical approval from the Regional Ethical Review Board at Lund University, Lund, Sweden (no. 398-01, LU 2014/146). The study followed the guidelines in the Declaration of Helsinki.

## Results

In total, 1047 out of 1543 eligible patients (68%) answered the HAQ and fulfilled the inclusion criteria. The mean age of survey respondents was 67 years (SD 13), 72% were women, and 53% reported HEPA levels.

There were no differences between non-respondents and those included in the study in age, at inclusion, mean 51.8 years (SD 15) vs. mean 51.1 years (SD 13), *P* = 0.4 or sex, 74% vs. 72% women, *P* = 0.5. Non-respondents were less often rheumatoid factor positive, 56% vs. 66%, *P* < 0.001. Please see Fig. 1.

### Better vs. worse physical function

Patients with better physical function (*n* = 511, 49%, HAQ ranging from 0 to 0.38) were younger, more often men, non-obese, and non-smokers, compared to patients with worse physical function (*n* = 536, 51%, HAQ ranging from 0.5 to 3.0) (Table 1). They also reported less pain and fatigue, better patient global assessment, health-related quality of life and empowerment, and fewer tender and swollen joints. The rate of comorbidities was lower in patients with better physical function (Table 1). There were differences in medical treatment: patients with worse physical function were treated with any DMARDs or CS to a greater extent, compared to patients with better physical function.

**Table 1:** Characteristics and comparison of individuals with worse physical function (PF), as indicated by a Health Assessment Questionnaire (HAQ) score of ≥ 0.5, versus those with better physical function (HAQ score < 0.5)

		Better PF Median (IQR)	Worse PF Median (IQR)	*P*-value
*n*		511	536	
Age, years		**67 (19)**	**71 (15)**	**< 0.001**
Sex, female, *n* (%)		**340 (66)**	**416 (78)**	**< 0.001**
RF-positive, *n* (%)		333 (67)	345 (65)	0.577
Disease duration, year		14 (6)	15 (7)	0.469
Obese (≥ 30 kg/m^2^), *n* (%)		**64 (13)**	**101 (20)**	**0.004**
Ever smoker,n (%)		**278 (55)**	**334 (63)**	**0.007**
HAQ, 0–3		**0 (0.25)**	**1.00 (0.63)**	**< 0.001**
NRS pain, 0–10		**2 (3)**	**5 (3)**	**< 0.001**
NRS fatigue, 0–10		**2 (5)**	**6 (4)**	**< 0.001**
NRS PatGA, 0–10		**1 (3)**	**4 (3)**	**< 0.001**
SJC, 0–28		**0 (2)**	**2 (6)**	**< 0.001**
TJC, 0–28		**1 (3)**	**5 (9)**	**< 0.001**
EQ5D, 0–1		**0.80 (0.20)**	**0.69 (0.14)**	**< 0.001**
SWE-RES-23, 1–5		**3.9 (1.1)**	**3.7 (4.0)**	**< 0.001**
SWE-RES-23, subscale 1		**4.0 (1.3)**	**3.8 (1.0)**	**< 0.001**
SWE-RES-23, subscale 2		**4.0** **(1.3)**	**3.8 (0.8)**	**< 0.001**
SWE-RES-23, subscale 3		**3.8 (1.5)**	**3.5 (1.0)**	**< 0.001**
SWE-RES-23, subscale 4		**4.0 (2.0)**	**3.7 (1.0)**	**< 0.001**
SWE-RES-23, subscale 5		**4.0 (2.0)**	**4.0 (1.5)**	**< 0.001**
Comorbidities	CVD, *n* (%)	**217 (42)**	**305 (58)**	**< 0.001**
	PD, *n* (%)	**47 (9)**	**85 (16)**	**0.001**
	T1D, *n* (%)	6 (1)	9 (2)	0.474
	T2D, n (%)	**31 (7)**	**60 (12)**	**0.003**
	Fibromyalgia, *n* (%)	**3 (1)**	**36 (7)**	**< 0.001**
	Mb. Crohn, *n* (%)	5 (1)	4 (1)	0.704
	Psoriasis, *n* (%)	23 (5)	36 (7)	0.121
	Depression, *n* (%)	**19 (4)**	**47 (10)**	**0.001**
Treatment	No DMARD/CS, *n* (%)	**146 (29)**	**114 (21)**	**< 0.001**
	cDMARD, *n* (%)	**222 (44)**	**212 (40)**	
	bDMARD, *n* (%)	**124 (24)**	**158 (30)**	
	Only CS, *n* (%)	**18 (4)**	**50 (9)**	

*CVD* cardiovascular disease, *CS* corticosteroids, *DMARD* disease-modifying anti-rheumatic drugs, *T1D* diabetes type 1, *T2D* diabetes type 2, *EQ5D* EuroQol five dimensions, *HAQ* Health Assessment Questionnaire, *NRS* Numeric Rating Scale, *PD* pulmonary disease, *SJC* swollen joint count, *SWE-RES-23* Swedish Rheumatic Disease Empowerment Scale-23 *TJC* tender joint count

Values in bold indicate significant statistical differences

### Reporting/not reporting HEPA in the group with better physical function

In the group with better physical function, 62% reported HEPA. Younger age, female sex, not being obese, being a non-smoker, and not reporting the comorbidity type II diabetes were associated with HEPA, also after adjusting for age and sex (Table 2). Less pain, fatigue, and better patient global assessment were also associated with HEPA, in both the univariate and the multivariate regression models (Tables 2 and 3). There were no differences in medical treatment.

**Table 2:** Associations with meeting the criteria for health-enhancing physical activity (HEPA) among individuals with *better* physical function were analysed using logistic regression models. Multivariate models were adjusted for age and sex

	Univariate model				Multivariate model			
	No.	OR	95% CI	*P*-value	OR	95% CI	*P*-value	
Age, years	511	**0.982**	**0.969–0.996**	**0.009**	**0.984**	**0.970–0.997**	**0.018**	
Sex, female	511	**1.489**	**1.022–2.168**	**0.038**	1.407	0.962–2.058	0.079	
RF positive	511	0.835	0.565–1.232	0.363				
Disease duration	511	1.006	0.959–1.056	0.795				
Obese (≥ 30 kg/m^2^)	499	**0.388**	**0.227–0.662**	**0.001**	**0.380**	**0.221–0.651**	**< 0.001**	
Ever smoker	506	**0.654**	**0.453–0.945**	**0.024**	0.703	0.484–1.022	0.065	
HAQ, 0–3	511	0.889	0.237–3.331	0.862				
NRS pain, 0–10	485	**0.915**	**0.843–0.994**	**0.035**	**0.905**	**0.832–0.985**	**0.020**	
NRS Fatigue, 0–10	489	**0.909**	**0.847–0.976**	**0.009**	**0.887**	**0.824–0.954**	**0.001**	
NRS PatGA, 0–10	494	**0.891**	**0.805–0.986**	**0.026**	**0.888**	**0.801–0.984**	**0.024**	
SJC, 0–28	511	0.972	0.921–1.026	0.308				
TJC, 0–28	511	1.006	0.964–1.049	0.797				
EQ5D, 0–1	489	2.323	0.687–7.855	0.175	3.004	0.866–10.42	0.083	
SWE-RES 23, 1–5	388	1.223	0.902–1.660	0.195	1.226	0.901–1.668	0.196	
SWE-RES23, subscale 1	420	**1.301**	**1.004–1.686**	**0.046**	**1.308**	**1.008–1.698**	**0.044**	
SWE-RES23, subscale 2	421	1.131	0.866–1.477	0.365				
SWE-RES23, subscale 3	424	1.010	0.800–1.276	0.933				
SWE-RES23, subscale 4	428	1.218	0.965–1.536	0.097	1.192	0.942–1.510	0.144	
SWE-RES23, subscale 5	438	1.132	0.938–1.367	0.195	1.153	0.954–1.394	0.142	
CVD	509	0.728	0.507–1.046	0.086	0.862	0.584–1.273	0.456	
PD	511	0.657	0.359–1.201	0.172	0.701	0.380–1.291	0.254	
T1D	470	1.135	0.206–6.263	0.884				
T2D	473	**0.328**	**0.155–0.693**	**0.004**	**0.373**	**0.174–0.800**	**0.011**	
Fibromyalgia	475	0.279	0.025–3.098	0.299				
Mb Crohn	464	0.364	0.060–2.199	0.271				
Psoriasis	476	1.310	0.528–3.249	0.561				
Depression	463	0.596	0.237–1.498	0.271				
cDMARD (%)	510	1.299	0.846–1.994	0.232	1.258	0.816–1.940	0.300	
bDMARD (%)	510	1.401	0.853–2.302	0.183	1.242	0.746–2.067	0.404	
Only CS (%)	510	0.718	0.269–1.914	0.507				

*CVD* cardiovascular disease, *CS* corticosteroids, *DMARD* disease-modifying anti-rheumatic drugs, *T1D* diabetes type 1, *T2D* diabetes type 2, *EQ5D* Euroqol five dimensions, *HAQ* Health Assessment Questionnaire, *HEPA* health-enhancing physical activity, *RF* rheumatoid factor, *NRS* Numeric Rating Scale, *PD* pulmonary disease, *SJC* swollen joint count, *SWE-RES-23* Swedish Rheumatic Disease Empowerment Scale-23, *TJC* tender joint count

Values in bold indicate significant statistical differences

**Table 3:** Characteristics of individuals with better physical function (HAQ < 0.5) who met the recommended health-enhancing physical activity levels (HEPA) compared to those who did not meet these recommendations (Non-HEPA) as assessed in the questionnaire

		HEPA Median (IQR)	Non-HEPA Median (IQR)	*P*-value
N		319	192	
Age, years		**66 (21)**	**69 (17)**	**0.006**
Sex, female, *n* (%)		**223 (70)**	**117 (61)**	**0.037**
RF positive, *n* (%)		204 (65)	129 (69)	0.363
Disease duration. year		15 (6)	14 (6)	0.820
Obese (≥ 30 kg/m^2^), *n* (%)		**27 (9)**	**37 (20)**	**< 0.001**
Ever smoker, *n* (%)		**163 (51)**	**115 (62)**	**0.023**
HAQ, 0–3		0.00 (0.25)	0.00 (0.25)	0.753
NRS pain, 0–10		**2 (3)**	**2 (4)**	**0.035**
NRS Fatigue, 0–10		**2 (4)**	**3 (4)**	**0.011**
NRS PatGA, 0–10		**1 (3)**	**2 (2)**	**0.045**
SJC, 0–28		0 (2)	0 (1)	0.345
TJC, 0–28		1 (3)	0 (4)	0.610
EQ5D, 0–1		0.80 (0.20)	0.80 (0.27)	0.187
SWE-RES 23, 1–5		4.0 (1.0)	3.9 (1.1)	0.189
SWE-RES23, subscale 1		4.0 (1.5)	4.0 (1.4)	0.050
SWE-RES23, subscale 2		4.0 (1.2)	4.0 (1.3)	0.411
SWE-RES23, subscale 3		3.8 (1.5)	3.8 (1.5)	0.751
SWE-RES23, subscale 4		4.0 (2.0)	4.0 (2.0)	0.157
SWE-RES23, subscale 5		4.0 (2.0)	4.0 (2.0)	0.216
Comorbidities	CVD (%)	126 (40)	91 (47)	0.085
	PD (%)	25 (8)	22 (11)	0.170
	T1D (%)	4 (1)	2 (1)	0.884
	T2D (%)	**12 (4)**	**19 (11)**	**0.002**
	Fibromyalgia, *n* (%)	1 (0.3)	2 (1)	0.267
	Mb Crohn, *n* (%)	2 (1)	3 (2)	0.251
	Psoriasis, *n* (%)	16 (5)	7 (4)	0.560
	Depression,*n* (%)	10 (3)	9 (6)	0.266
Treatment	No DMARD^/^CS (%)	27	32	0.336
	cDMARD (%)	45	41	
	bDMARD (%)	26	22	
	Only CS (%)	3	5	

*CVD* cardiovascular disease, *CS* corticosteroids, *DMARD* disease-modifying anti-rheumatic drugs, *T1D* diabetes type 1, *T2D* diabetes type 2, *EQ5D* Euroqol five dimensions, *HAQ* Health Assessment Questionnaire, *RF* rheumatoid factor, *NRS* Numeric Rating Scale, *PD* pulmonary disease, *SJC* swollen joint count, *SWE-RES-23* Swedish Rheumatic Disease Empowerment Scale-23, *TJC* tender joint count

Values in bold indicate significant statistical differences

### Reporting/not reporting HEPA in the group with worse physical function

In the group with worse physical function, 44% reported HEPA. In this group, younger age, not being obese, reporting less pain and fatigue, better patient global assessment, and fewer swollen and tender joints were associated with fulfilling HEPA after adjusting for age and sex. The absence of comorbidities, such as cardiovascular disease and type II diabetes, was associated with fulfilling HEPA. Better health-related quality of life and better empowerment scores were also associated with HEPA after age and sex adjustment. When analysing the subscales in SWE-RES-23 separately, the subscale focusing on self-management was associated with HEPA after adjustment for age and sex. Medical treatment with bDMARD was associated with fulfilling HEPA in the univariate model, but not after adjustment for age and sex (Tables 4 and 5).

**Table 4:** Characteristics of those with *worse* physical function who fulfilled recommended criteria for health-enhancing physical activity (HEPA), compared to those who did not fulfil recommended physical activity (Non-HEPA) in the questionnaire

		HEPA Median (IQR)	Non-HEPA Median (IQR)	*P*-value
*n*		234	302	
Age, years		**68 (15)**	**73 (14)**	**< 0.001**
Sex, Female, *n* (%)		184 (79)	232 (77)	0.618
RF positive, *n* (%)		150 (66)	195 (65)	0.904
Disease duration. year		15 (7)	15 (6)	0.474
Obese (≥ 30 kg/m^2^), *n* (%)		**34 (15)**	**67 (23)**	**0.014**
Ever smoker, *n* (%)		140 (60)	194 (66)	0.197
HAQ, 0–3		**0.88 (0.50)**	**1.13 (0.88)**	**< 0.001**
NRS pain, 0–10		**4 (4)**	**5 (4)**	**0.036**
NRS Fatigue, 0–10		5 (4)	6 (3)	0.064
NRS PatGA, 0–10		4 (3)	5 (3)	0.066
SJC, 0–28		2 (5)	2 (7)	0.189
TJC, 0–28		5 (8)	5 (11)	0.255
EQ5D, 0–1		**0.73 (0.18)**	**0.66 (0.23)**	**< 0.001**
SWE-RES 23, 1–5		**3.7 (0.8)**	**3.6 (0.8)**	**0.023**
SWE-RES23, subscale 1		**4.0 (1.0)**	**3.8 (0.83)**	**0.040**
SWE-RES23, subscale 2		**4.0 (1.0)**	**3.8 (1.0)**	**0.034**
SWE-RES23, subscale 3		3.5 (1.0)	3.2 (1.0)	0.214
SWE-RES23, subscale 4		**3.7 (1.2)**	**3.7 (1.0)**	**0.007**
SWE-RES23, subscale 5		4.0 (1.5)	4.0 (1.0)	0.799
Comorbidities	CVD, *n* (%)	**112 (48)**	**193 (65)**	**< 0.001**
	PD, *n* (%)	**32 (14)**	**53 (18)**	**0.023**
	T1D, *n* (%)	4 (2)	5 (2)	0.960
	T2D, *n* (%)	**17 (8)**	**43 (16)**	**0.006**
	Fibromyalgia, *n* (%)	17 (8)	19 (7)	0.788
	Mb Crohn, *n* (%)	3 (1)	1 (0)	0.236
	Psoriasis, *n* (%)	15 (7)	21 (8)	0.658
	Depression, *n* (%)	24 (11)	23 (9)	0.416
Treatment	No DMARD^/^CS, *n* (%)	**42 (18)**	**72 (24)**	**0.006**
	cDMARD, *n* (%)	**89 (38)**	**123 (41)**	
	bDMARD, *n* (%)	**86 (37)**	**42 (24)**	
	Only CS, *n* (%)	**16 (7)**	**34 (11)**	

*CVD* cardiovascular disease, *CS* corticosteroids, *DMARD* disease-modifying anti-rheumatic drugs, *T1D* diabetes type 1, *T2D* diabetes type 2, *EQ5D* Euroqol five dimensions, *HAQ* Health Assessment Questionnaire, *HEPA* health-enhancing physical activity, *NRS* Numeric Rating Scale, *PD* pulmonary disease, *SJC* swollen joint count, *SWE-RES-23* Swedish Rheumatic Disease Empowerment Scale-23, *TJC* tender joint count

Values in bold indicate significant statistical differences

**Table 5:** Associations with fulfilling criteria for HEPA in those with *worse* physical function, analysed with logistic regression models. Multivariate models were adjusted for age and sex

	Univariate model				Multivariate model		
	No.	OR	95% CI	*P*-value	OR	95% CI	*P*-value
Age, years	536	**0.959**	**0.944–0.974**	**< 0.001**	**0.958**	**0.943–0.973**	**< 0.001**
Sex, female	536	1.110	0.736–1.675	0.618			
RF positive	536	1.022	0.712–1.467	0.904			
Disease duration	536	1.018	0.975–1.064	0.410			
Obese (≥ 30 kg/m^2^)	516	**0.567**	**0.360–0.894**	**0.015**	**0.519**	**0.324–0.830**	**0.006**
Ever smoker	529	0.791	0.555–1.129	0.197	0.754	0.519–1.097	0.140
HAQ, 0–3	536	**0.288**	**0.193–0.429**	**< 0.001**	**0.315**	**0.208–0.476**	**< 0.001**
NRS pain, 0–10	520	**0.857**	**0.793–0.926**	**< 0.001**	**0.838**	**0.772–0.909**	**< 0.001**
NRS fatigue, 0–10	522	**0.912**	**0.851–0.977**	**0.009**	**0.883**	**0.821–0.950**	**0.001**
NRS PatGA, 0–10	519	**0.871**	**0.804–0.942**	**0.001**	**0.848**	**0.780–0.921**	**< 0.001**
SJC, 0–28	536	**0.959**	**0.929–0.990**	**0.009**	**0.958**	**0.927–0.991**	**0.012**
TJC, 0–28	536	**0.972**	**0.948–0.995**	**0.019**	**0.971**	**0.947–0.996**	**0.023**
EQ5D, 0–1	517	**7.501**	**3.432–16.40**	** < 0.001**	**8.355**	**3.745–18.64**	**< 0.001**
SWE-RES 23, 1–5	440	**1.428**	**1.050–1.944**	**0.023**	**1.042**	**1.019–1.928**	**0.038**
SWE-RES23, subscale 1	473	**1.338**	**1.031–1.7.7**	**0.029**	1.299	0.991–1.701	0.058
SWE-RES23, subscale 2	475	**1.334**	**1.041–1.709**	**0.023**	**1.332**	**1.030–1.723**	**0.029**
SWE-RES23, subscale 3	474	1.200	0.955–1.509	0.118	1.178	0.931–1.489	0.172
SWE-RES23, subscale 4	475	**1.428**	**1.120–1.820**	**0.004**	1.279	0.993–1.646	0.056
SWE-RES23, subscale 5	484	0.970	0.829–1.136	0.708			
CVD	530	**0.508**	**0.358–0.721**	**< 0.001**	**0.649**	**0.446–0.946**	**0.025**
PD	536	0.744	0.462–1.198	0.224	0.726	0.444–1.188	0.203
T1D	486	0.967	0.256–3.644	0.960			
T2D	498	**0.441**	**0.244–0.798**	**0.007**	**0.529**	**0.289–0.969**	**0.039**
Fibromyalgia	497	1.098	0.556–2.166	0.788			
Mb Crohn	478	3.614	0.373–34.99	0.267			
Psoriasis	500	0.856	0.431–1.703	0.659			
Depression	484	1.283	0.703–2.343	0.417			
cDMARD	212	1.240	0.777–1.981	0.367			
bDMARD	158	**2.048**	**1.251–3.352**	**0.004**	1.570	0.939–2.626	0.086
Only CS	50	0.807	0.398–1.634	0.551			

*CVD* cardiovascular disease, *CS* corticosteroids, *DMARD* disease-modifying anti-rheumatic drugs, *T1D* diabetes type 1, *T2D* diabetes type 2, *EQ5D* Euroqol five dimensions, *HAQ* Health Assessment Questionnaire, *HEPA*, health-enhancing physical activity, *NRS*, Numeric Rating Scale, *PD* pulmonary disease, *SJC* swollen joint count, *SWE-RES-23* Swedish Rheumatic Disease Empowerment Scale-23, *TJC* tender joint count

Values in bold indicates significant statitical differences

## Discussion

The present study assessed factors associated with HEPA in patients with RA, stratified by physical function. Several disease-related factors were associated with HEPA, irrespective of physical function. However, in patients with worse physical function, factors such as health-related quality of life and empowerment were associated with HEPA and may represent factors important to address when tailoring physical activity interventions for this group of patients.

A large proportion of the patients did not reach recommended levels of physical activity to enhance health, which is in accordance with earlier studies [18–21]. The positive association between physical function, physical activity level, and health-related quality of life in patients with RA is well known [18, 22–24]. Physical function, health-related quality of life, and disease-related factors, such as pain, fatigue, and painful and swollen joints, show a bi-directional relationship with PA, where they may act as barriers to engaging in physical activity and—also—they may improve with exercise [19, 25–30]. The multi-faceted relationship between physical function, disease-related factors, health-related quality of life, patient empowerment, and HEPA calls for a multidisciplinary awareness and clinical interventions to support healthy lifestyle choices. Interventions targeting quality of life and its multifaceted dimensions may improve both the motivation for physical activity and the patients’ perceived health-related quality of life.

### Empowerment and HEPA

In the last decades, there has been an increased focus on the importance of facilitating physical activity and exercise in patients with RA, both as health promotion and as disease prevention. The increased risk for cardiovascular diseases in this population and the evidence that physical activity can improve disease outcomes make it even more important [21, 31]. Some factors associated with HEPA in the present study were similar, irrespective of the level of physical function, while we did find some distinctions between the stratified groups. Patients with worse physical function, who reported HEPA, had better empowerment scores, compared with those who did not fulfil HEPA, a result that was not found in the group with better physical function. The facilitation of empowerment when tailoring interventions aimed at improving physical activity levels seems to be of great importance, especially if the patients are challenged by impaired physical function.

Empowerment is a measure of an individual’s perceived control, and in a health context, it relates to the individual’s competence in managing their own health and health care [32, 33]. Health professionals’ facilitation role is to educate, communicate with, and support patients to self-manage a proper level of physical activity, where empowerment is a key factor in self-management [33, 34]. Empowerment may work as a facilitator for engaging in physical activity, which, in turn, may enhance a person’s experience of being empowered, in a bi-directional relationship [35].

We used the SWE-RES-23 to measure empowerment in patients with established RA [15]. Higher scores in the subscales goal achievement, self-knowledge, and readiness for change were all associated with HEPA in the group of patients with worse physical function, according to the univariate analysis. The ability to set and achieve goals, having good self-knowledge, and willingness to change are indicators of patient empowerment supporting patients’ behaviour, such as participating in shared decision-making and self-management. Empowered patients can better adapt to a chronic illness, show better health-related quality of life and satisfaction with life, and promote independence [33].

Programmes that target the multifaceted relationship between physical function and HEPA, while considering the individual participants’ context, may enhance both the motivation for physical activity and participants’ health and quality of life.

#### Lifestyle and HEPA

In the current study, obese patients, smokers, and patients with type II diabetes were less likely to report HEPA, irrespective of their physical function level, and they require special attention in the clinic. This is in line with the findings of a previous study that shows that every second patient with RA had two or more unhealthy lifestyle factors, of which being overweight and not fulfilling HEPA were the two most common [36]. Similar findings have been reported in the general population [37]. The prevalence of metabolic syndrome is rising, including among patients with RA [38], exacerbating the risk for cardiovascular diseases [39]. Smoking is also a risk factor for cardiovascular diseases and a worse disease outcome in RA [40]. This indicates the importance of education, discussion, support, and empowerment of patients to optimise their ability to make healthy lifestyle choices.

### Limitations

Given the cross-sectional design of this study, we cannot establish a cause-effect relationship between physical function, health-related quality of life, empowerment, and HEPA.

All physical activity data in the present study were self-reported, and the correlation between self-reported data and objectively assessed aerobic capacity or physical activity measured with accelerometers is low [41]. However, in a questionnaire survey of the size of that in the current study, self-reporting is the only option. Self-reported anthropometric measures and disease-related measures, such as swollen and tender joint count, performed by health professionals and patients are reported to have an acceptable correlation [42].

## Conclusion

The present study assessed factors associated with HEPA in patients with RA, stratified by physical function. Several disease-related factors were associated with HEPA, irrespective of physical function. However, in patients with worse physical function, factors such as health-related quality of life and empowerment were associated with HEPA. The results indicate a complex relationship between various factors that can affect the possibility of being physically active while living with RA. It may be important to address patients’ health-related quality of life and facilitate empowerment when tailoring physical activity interventions in the clinic, especially for patients with impaired physical function.

## Data Availability

The data supporting this study’s findings are available upon reasonable request to the corresponding author.
